# Predictors for short-term successful weaning from continuous renal replacement therapy: a systematic review and meta-analysis

**DOI:** 10.1080/0886022X.2023.2176170

**Published:** 2023-02-10

**Authors:** Yu Li, Xiaoqi Deng, Jiaxing Feng, Bo Xu, Yulei Chen, Zhanying Li, Xiaodan Guo, Tianjun Guan

**Affiliations:** a Department of Nephrology, Zhongshan Hospital affiliated to Xiamen University, Xiamen, China; b Department of Gastroenterology, Zhongshan Hospital affiliated to Xiamen University, Xiamen, China; c School of Medicine, Xiamen University, Xiamen, China

**Keywords:** Continuous renal replacement therapy, weaning, discontinuation, predictor, acute kidney injury, meta-analysis

## Abstract

The systemic review and meta-analysis aimed to identify the predictors for short-term successful weaning from CRRT in severe AKI patients. PubMed, Embase, the Cochrane Library, and grey literature were searched for relevant studies investigating variables for short-term successful weaning from CRRT to August 2022. Our criteria included patients with AKI who required CRRT but excluded patients with kidney failure. The pooled odds ratios (OR) and 95% confidence intervals (CI) were calculated using fixed-effect (I^2^≤50% and P-value of the Q statistic > 0.1) or random-effect models (I^2^>50% or p-value of the Q statistic ≤ 0.1) as appropriate. Our search yielded 11 studies and described 11 variables. The pooled analysis showed that chronic kidney disease (OR = 0.638, 95% CI: 0.491–0.829), CRRT duration (OR = 0.913, 95% CI: 0.882–0.946), and urine output at the cessation of CRRT (per 100 mL/day increase) (OR = 1.084, 95% CI: 1.061–1.108) were predictive factors for short-term successful weaning from CRRT. Male (OR = 0.827, 95% CI: 0.627-1.092), diabetes mellitus (OR = 0.970, 95% CI: 0.761–1.237), and sepsis (OR = 0.911, 95% CI: 0.717–1.158) were unrelated to the short-term weaning from CRRT. The relationship between hypertension, use of vasopressors or inotropes at the starting of CRRT, use of vasopressors or inotropes at the cessation of CRRT, use of diuretics at the cessation of CRRT, serum creatinine at the cessation of CRRT, and short-term weaning from CRRT remains unclear. Additional prospective studies are needed to evaluate this relationship further.

## Background

Acute kidney injury (AKI) is common and associated with significant mortality in critically ill patients [1]. The incidence of AKI in critically ill patients varies widely depending on the underlying disease, ranging from 6% to 67% [2–6]. Continuous renal replacement therapy (CRRT) has become the preferred mode over intermittent renal replacement therapy in severe AKI patients who required kidney replacement therapy (KRT) due to the hemodynamic stability and steady solute control [7]. Recently, the concept of CRRT trauma has emerged, referring to the occurrence of CRRT-related harmful adverse events [8,9]. CRRT can be detrimental for patients due to physical restriction, anti-coagulant administration, unexpected drug removal, and dialysis-related complications [10–14]. Therefore, it is important to recognize patients who may be early weaning from CRRT. At present, CRRT practices vary among clinicians and institutions, and there are some data regarding the optimal commencement and dosing of CRRT [15–18]. However, less is known about the discontinuation criteria for CRRT. The primary objective of this systemic review and meta-analysis was to identify predictors for short-term successful weaning from CRRT in severe AKI patients.

## Methods

### Search strategy

The protocol of this study was registered with the International Prospective Register of Systematic Reviews (PROSPERO CRD 42021272492). We followed the Preferred Reporting Items for Systemic Reviews and Meta-analyses (PRISMA) checklist [19] for reporting. PubMed, Embase, and the Cochrane Library were systematically searched for relevant studies investigating variables for short-term successful weaning from CRRT from database inception to August 2022 without language restrictions. Subject words and free words, including ‘continuous renal replacement therapy’, ‘weaning’, ‘discontinuation’, ‘cessation’, ‘risk’, and ‘predict’, were combined during study retrieval. The complete search strategies used for the major databases are presented in Additional file 1. The following grey literature databases also were searched: GreyNet International, SIGLE (The System for Information on Grey Literature in Europe), Open Gery, and Gery Literature Report. Further, the relevant studies' references were examined for more studies.

### Inclusion and exclusion criteria

**Figure 1.**
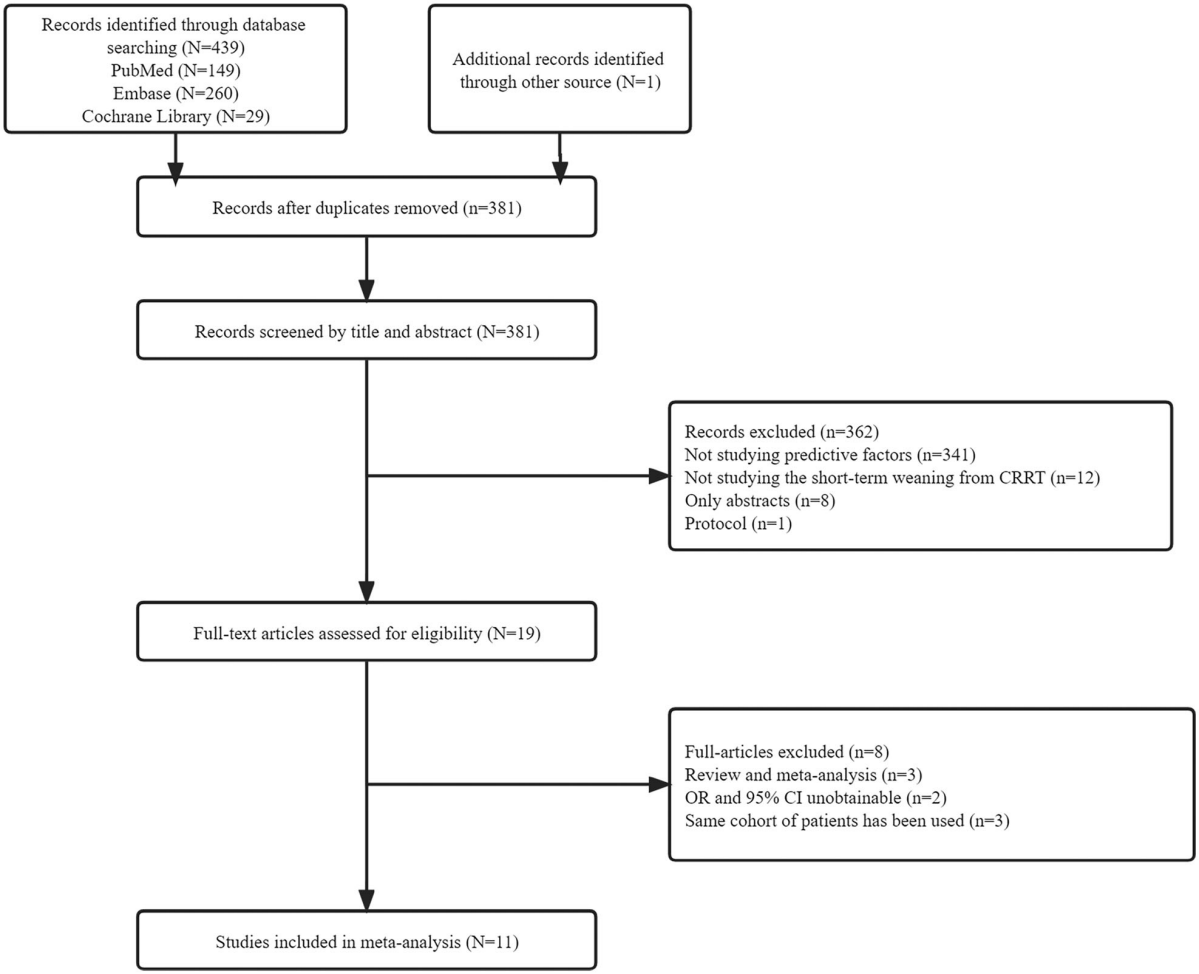
Flow chart of included studies.

Studies with prospective, cross-sectional or retrospective design will be included with the following inclusion criteria: (I) the study that reported predictive factors for short-term successful weaning from CRRT in severe AKI patients in all countries and regions; (II) patients were divided based on the KRT requirement in the short term after cessation of CRRT; and (III) the CRRT cycle was completely terminated (CRRT device disassembled) rather than temporarily stopped (e.g., for diagnostic or therapeutic measures outside the intensive care unit). The specific exclusion criteria are detailed in Figure 1.

### Data extraction and quality assessment

The following data were recorded independently by two independent researchers (YL and XD): general characteristics of eligible studies, the definition of short-term successful weaning from CRRT, and any possible predictive factors for successful weaning from CRRT (e.g., demographics, clinical and laboratory variables at the starting or the cessation of CRRT). The multivariate-adjusted odds ratios (OR) and 95% confidence intervals (CI) were extracted if available, otherwise, unadjusted ORs and 95% CIs were calculated. Newcastle-Ottawa Scale (NOS) was used to evaluate the study quality [20]. In case of discrepancies over-extraction of data or scoring of the study quality, a third person (TG) was involved and the results were discussed until a consensus was reached.

### Statistical analysis

Stata 16.0 software was used for statistical analysis. Inter-rater reliability was assessed using Cohen’s kappa statistics. Statistical heterogeneity among studies was assessed by using the *p*-value of the Q statistic and I^2^ statistic. The pooled ORs were calculated using the fixed-effects model if the heterogeneity was acceptable (I^2^≤50% and *p*-value of the Q statistic > 0.1), otherwise, a random-effects model was used adopted (I^2^≥50% or *p*-value of the Q statistic ≤ 0.1). Subgroup analysis and sensitivity analysis were conducted to find the source of heterogeneity. Publication bias was assessed with the funnel plot and Egger’s test when adequate studies were included. When asymmetry of the funnel plots was present, the trim-and-fill method will be further applied.

## Results

### Study characteristics

Out of 439 citations, 11 studies [21–31] with a total of 2741 patients who fulfilled the inclusion and exclusion criteria were enrolled in the study. The study flowchart is presented in Figure 1. The general characteristics of the included studies and quality scores are listed in Table 1. The definition of short-term successful weaning from CRRT is free from KRT for 7 or 14 days after the cessation of CRRT for most of the studies. The quality scores of included studies ranged from 6–9, with a median score of 8. The value of Cohen’s kappa is 0.866, indicating good consistency for the selection and data extraction of two independent researchers.

**Table 1.** General characteristics of studies included in the meta-analysis.

Study	Country	Study design	No. of total patients	No. of patients successful weaning from CRRT	Definition of successful weaning from CRRT	NOS score
Uchino, 2009	Multiple	PC	529	313	Free from CRRT for 7 days after stopping CRRT	7
Frohlich, 2012	Ireland	RC	85	53	Free from CRRT for 7 days after stopping CRRT	6
Heise, 2012	Germany	RC	225	103	Free from CRRT for at least 12 h, and discharged from ICU with no further RRT during hospital stay	9
Katayama, 2016	Japan	RC	116	97	Free from CRRT for 7 days after stopping CRRT	8
Han, 2016	Korea	RC	160	Not specified	Free from CRRT for 14 days after stopping CRRT	7
Raurich, 2018	Spain	RC	86	67	Free from CRRT for at least 12 h, and discharged from ICU with no further RRT during hospital stay	9
Kim, 2018	Korea	PC	110	89	Free from CRRT for 14 days after stopping CRRT	8
Jeon, 2018	Korea	PC	1176	517	Free from CRRT for 3 days after stopping CRRT	9
Chen, 2019	China	PC	110	78	Free from CRRT for 7 days after stopping CRRT	9
Stads, 2019	Netherlands	PC	92	61	Free from CRRT for 7 days after stopping CRRT	9
Yoshida, 2019	Japan	RC	52	38	Neither resuming CRRT for the next 48 h nor receiving IHD within 7 days after stopping CRRT	8

CRRT: continuous renal replacement therapy; IHD: intermittent hemodialysis; NOS: Newcastle-Ottawa Scale; PC, prospective cohort; RC: retrospective cohort.

### Predictors for short-term successful weaning from CRRT

#### Gender

**Figure 2.**
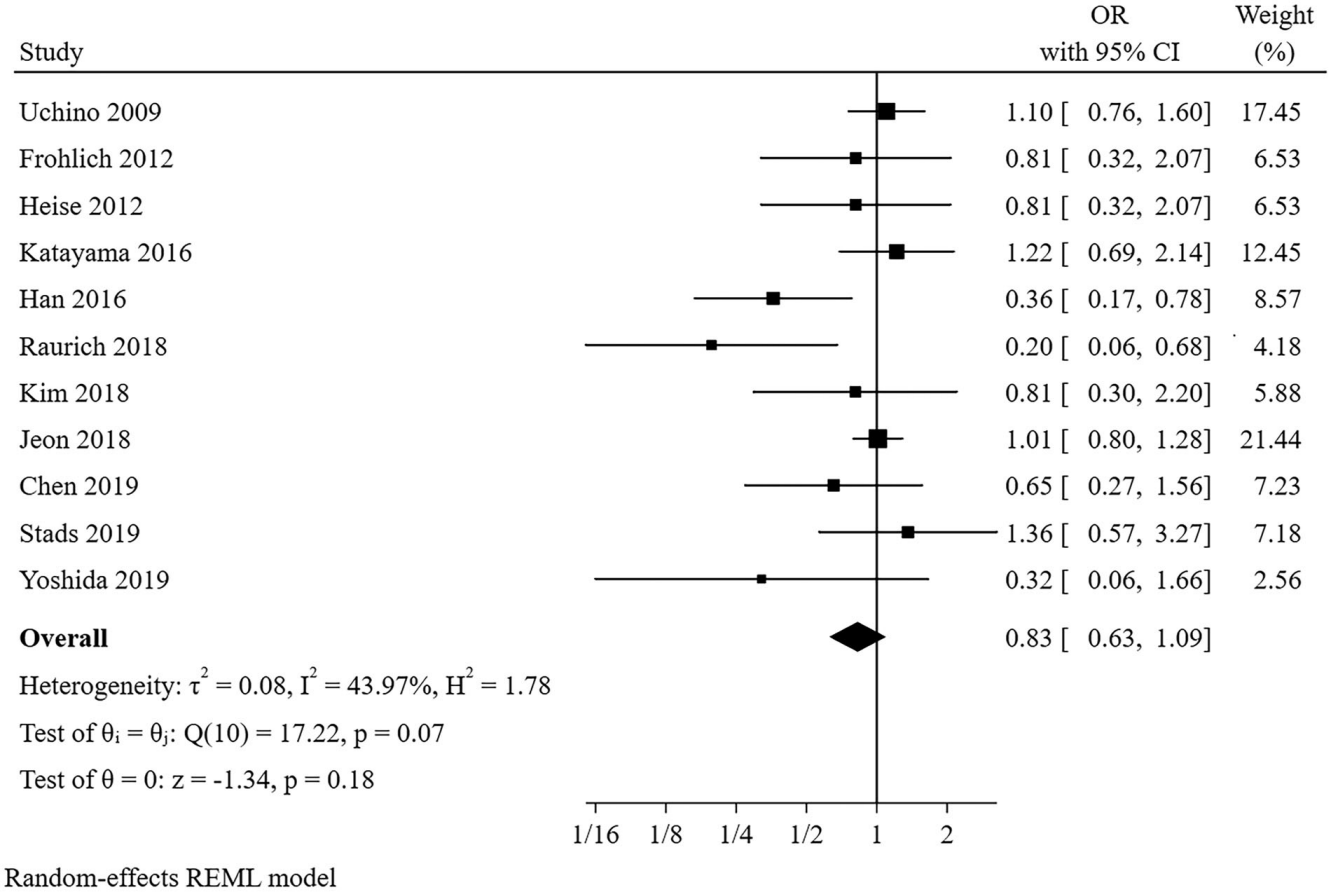
The forest plot showed the relationship between gender and successful weaning from CRRT.

Eleven studies [21–31] were included in the meta-analysis for gender (Figure 2). The result indicated that male was not a predictor for short-term successful weaning from CRRT (pooled OR = 0.827, 95% CI: 0.627–1.092, I^2^=43.97%, *p* = 0.070). Sensitivity analysis suggested that the pooled conclusion was robust. The funnel plot indicated publication bias (*p* = 0.017 for Egger’s test) (Additional file 2). After filling four studies, the funnel plot became symmetrical (Additional file 3), and the result barely changed for gender (pooled OR = 1.027, 95% CI: 0.713–1.487).

#### Hypertension

**Figure 3.**
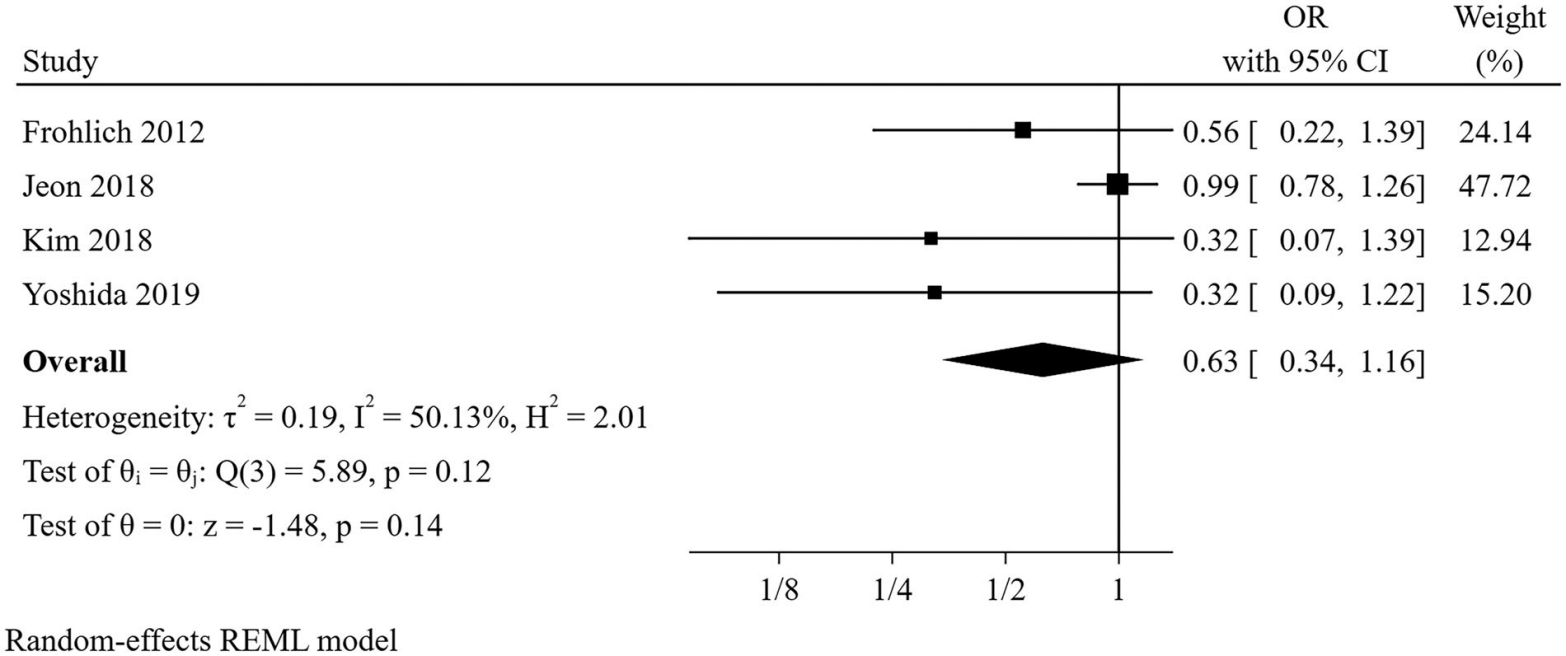
The forest plot showed the relationship between hypertension and successful weaning from CRRT.

Four studies [22,26,27,31] were included in the meta-analysis for hypertension (Figure 3). The result suggested that the presence of hypertension was not a predictor for short-term successful weaning from CRRT (pooled OR = 0.628, 95% CI: 0.339-1.162). However, there was significant heterogeneity (I^2^=50.13%, *p* = 0.117). Because the definition of short-term successful weaning from CRRT was 3 days in the study of Jeon and colleagues, which differed from 7 or 14 days in most of the included studies, we excluded this study in the sensitivity analysis. After this step, the heterogeneity completely disappeared (I^2^=0%, *p* = 0.725), and the pooled result altered (pooled OR = 0.432, 95% CI: 0.211–0.843), suggesting the pooled conclusion was not robust.

#### Diabetes mellitus

**Figure 4.**
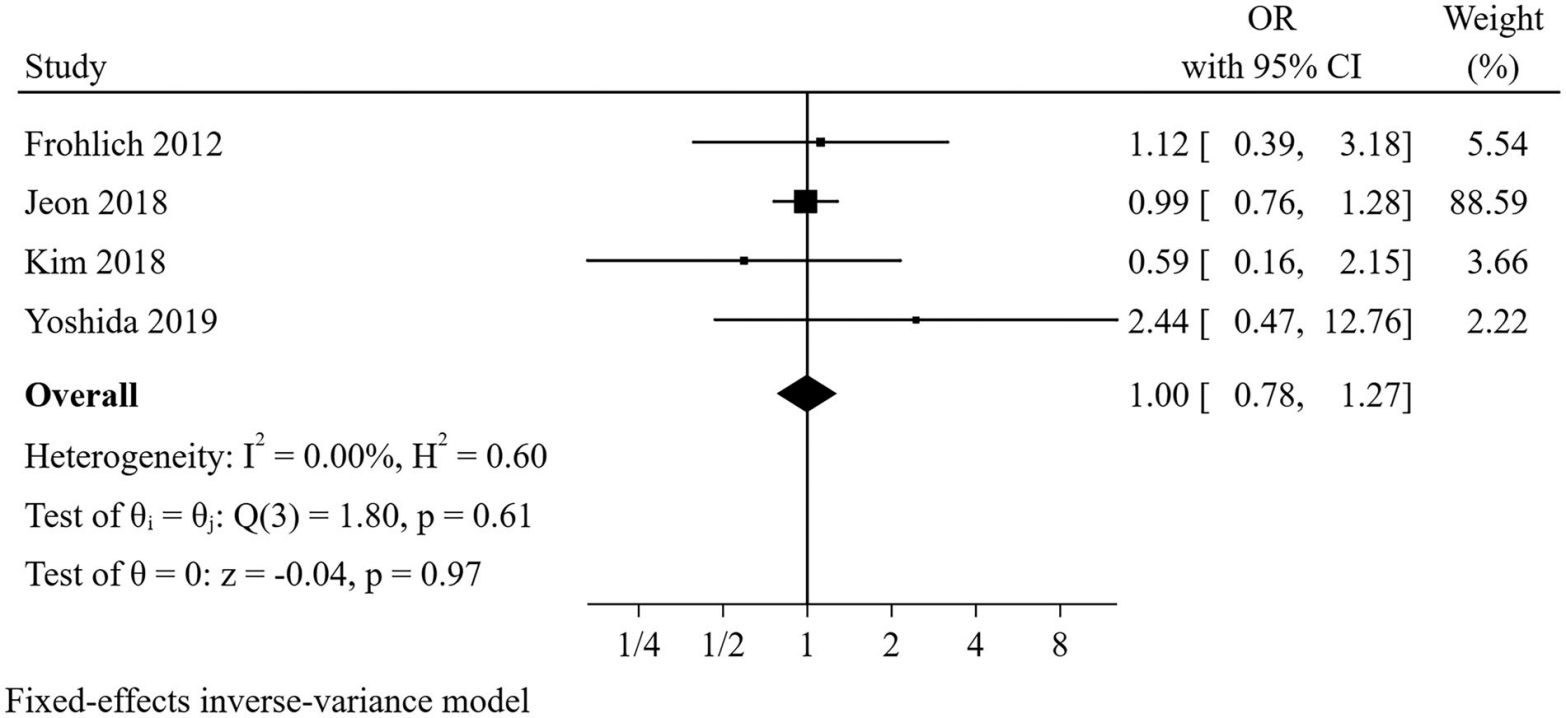
The forest plot showed the relationship between diabetes mellitus and successful weaning from CRRT.

Four studies [22,26,27,31] were included in the meta-analysis for diabetes millitus (Figure 4). The result indicated that the presence of diabetes mellitus was not a predictor for short-term successful weaning from CRRT (pooled OR = 0.995, 95% CI: 0.778–1.273, I^2^=0%, *p* = 0.614). Sensitivity analysis suggested that the pooled conclusion was robust.

#### Chronic kidney disease

**Figure 5.**
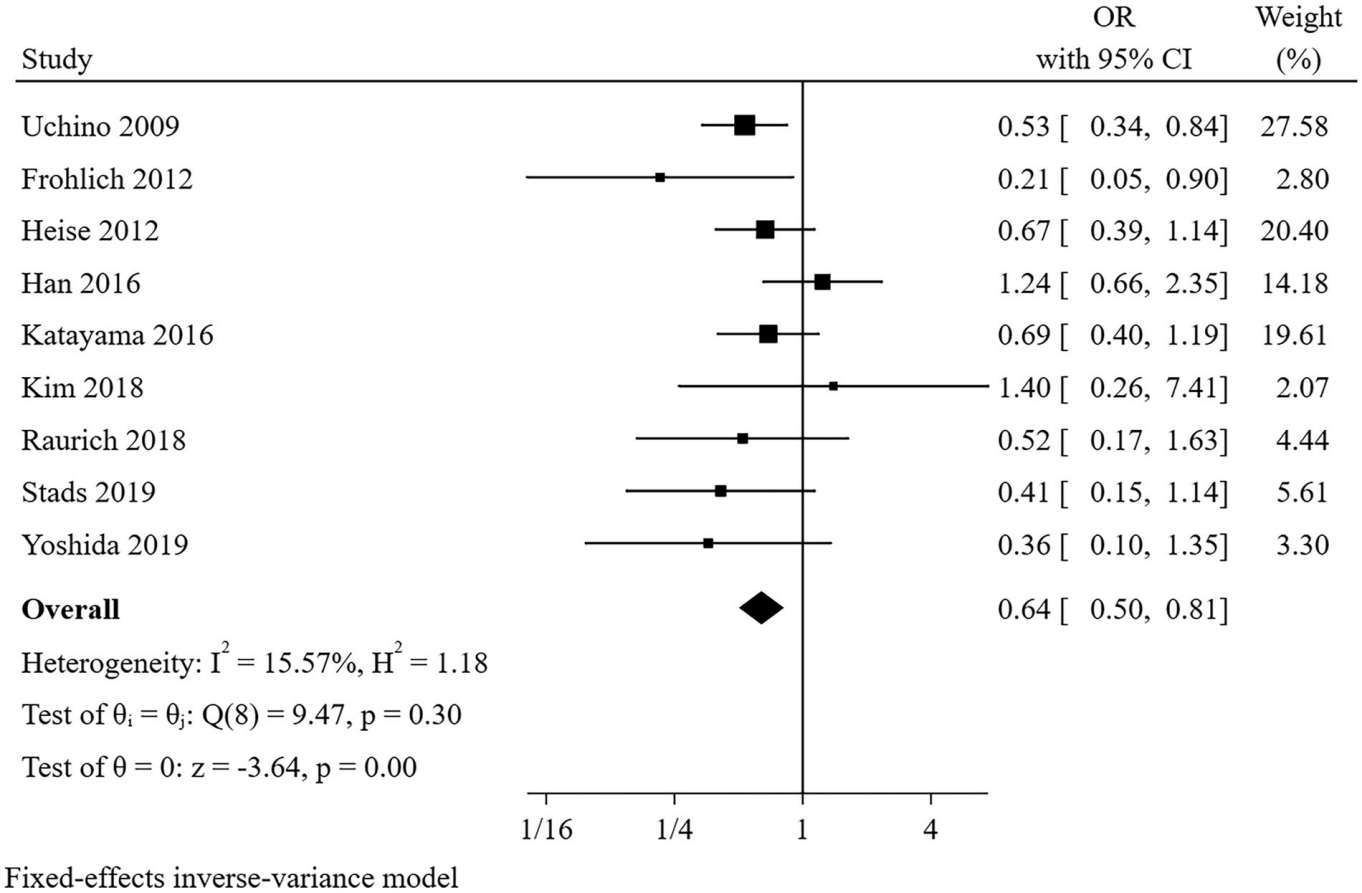
The forest plot showed the relationship between chronic kidney disease and successful weaning from CRRT.

Nine studies [21–25,27,28,30,31] were included in the meta-analysis for chronic kidney disease (CKD) (Figure 5). The result suggested that the presence of chronic kidney disease was a predictor for short-term successful weaning from CRRT (pooled OR = 0.638, 95% CI: 0.491–0.829, I^2^=15.57%, *p* = 0.304). Sensitivity analysis suggested that the pooled conclusion was robust. The funnel plot suggested that there was no publication bias (*p* = 0.017 for Egger’s test) (Additional file 4).

#### Sepsis

**Figure 6.**
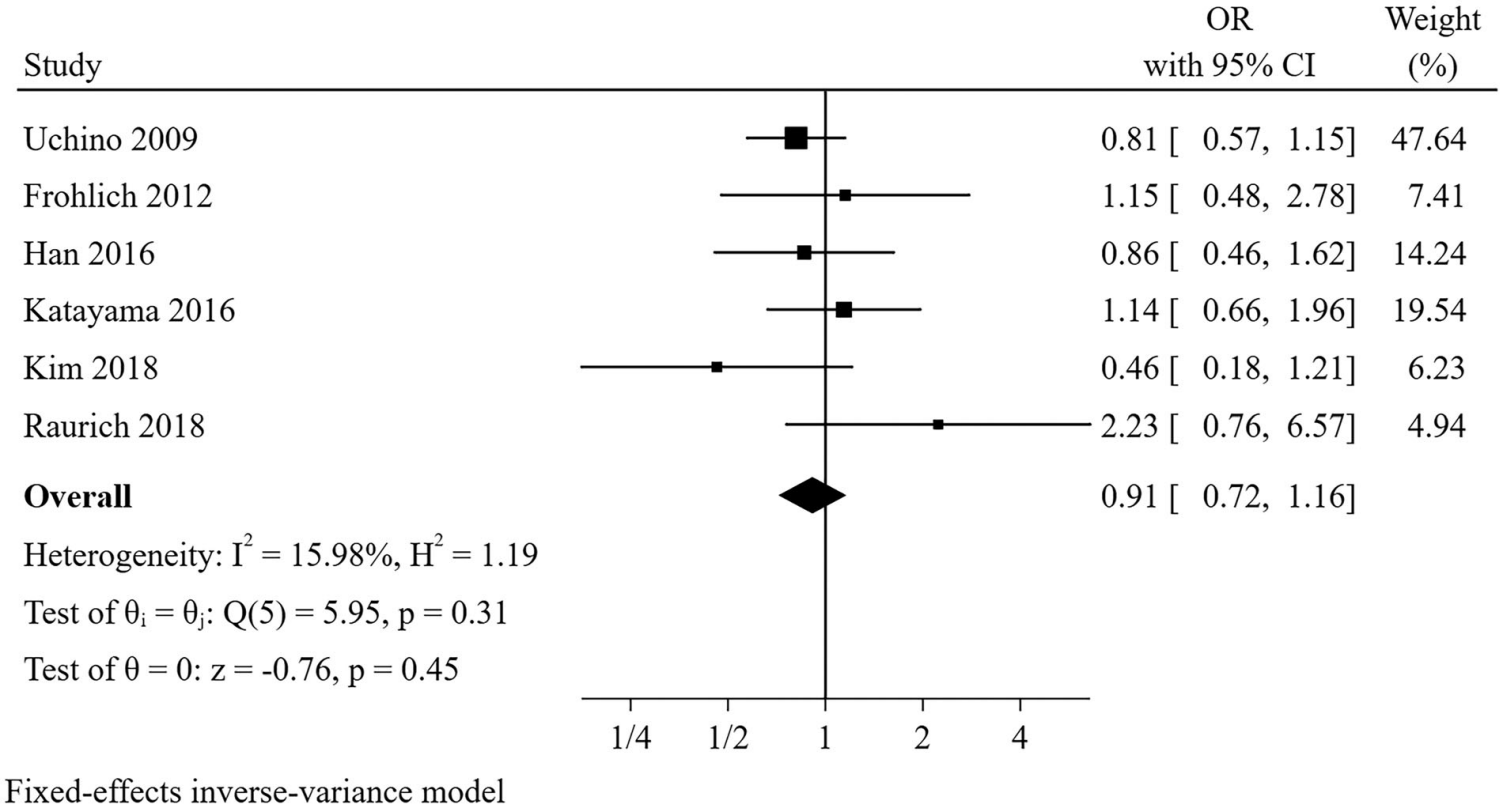
The forest plot showed the relationship between sepsis and successful weaning from CRRT.

Six studies [21,22,24,25,27,28] were included in the meta-analysis for patients with sepsis (Figure 6). The result showed that the presence of sepsis was not a predictor for short-term successful weaning from CRRT (pooled OR = 0.911, 95% CI: 0.717–1.158, I^2^=15.98%, *p* = 0.311). Sensitivity analysis suggested that the pooled conclusion was robust.

#### CRRT duration

**Figure 7.**
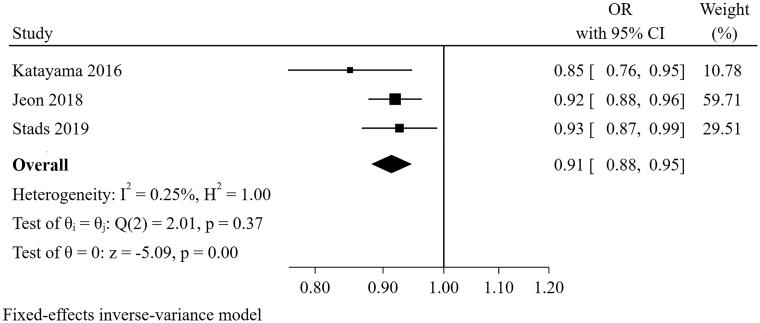
The forest plot showed the relationship between CRRT duration and successful weaning from CRRT.

Three studies [25, 26, 30] were included in the meta-analysis for CRRT duration (Fig. 7). The result suggested that the total number of days on CRRT was a predictor for short-term successful weaning from CRRT (pooled OR = 0.913, 95% CI: 0.882-0.946, I^2^=0.25%, *p* = 0.367). Sensitivity analysis suggested that the pooled conclusion was robust.

#### Use of vasopressor or inotrope at the starting of CRRT

**Figure 8.**
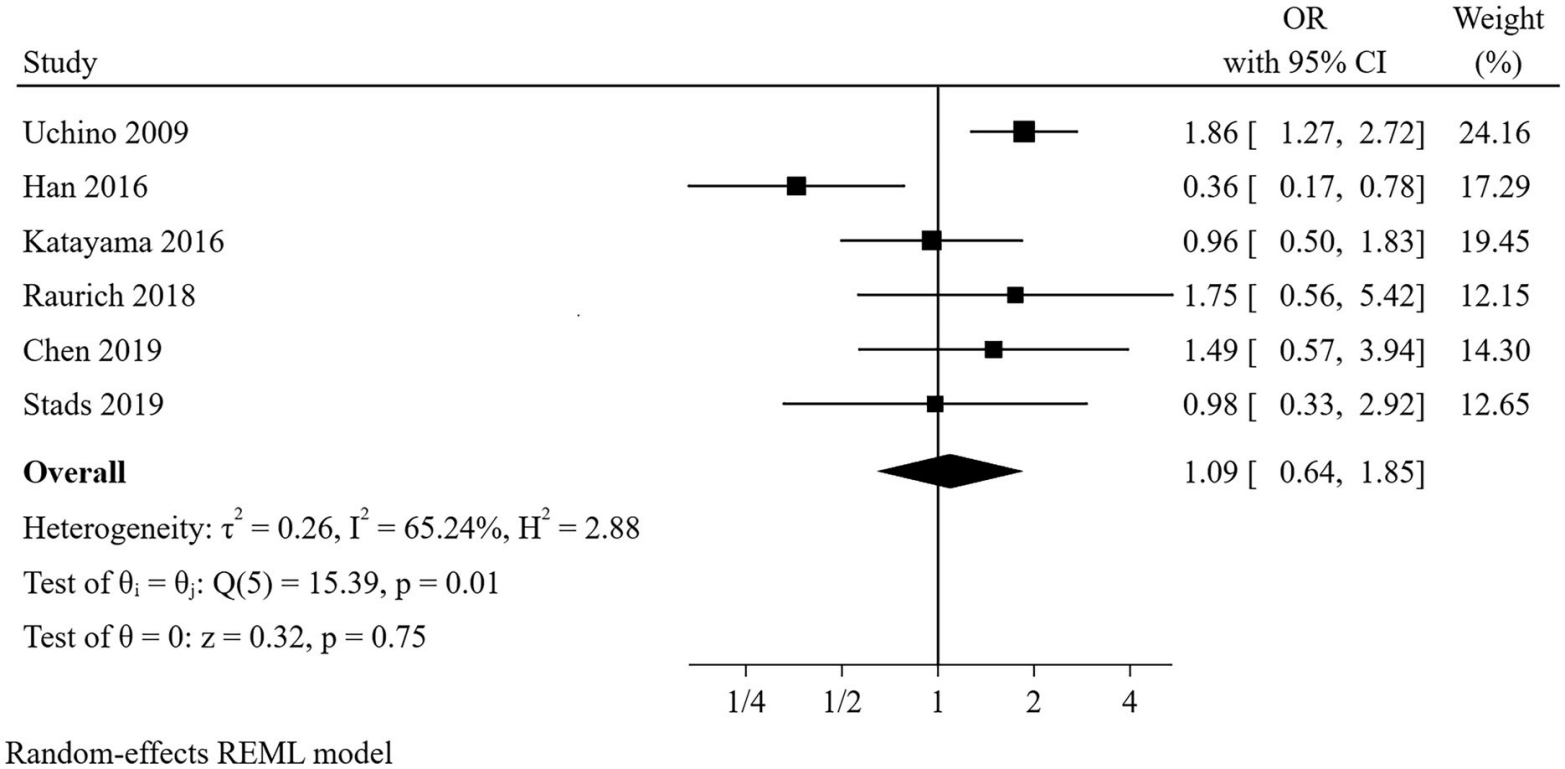
The forest plot showed the relationship between use of vasopressor or inotropes at the starting of CRRT and successful weaning from CRRT.

Six studies [21,24,25,28–30] were included in the meta-analysis for use of vasopressor or inotrope at the starting of CRRT (Figure 8). The result suggested that the use of vasopressor or inotrope at the starting of CRRT was not a predictor for short-term successful weaning from CRRT (pooled OR = 1.091, 95% CI: 0.644–1.894). However, there was significant heterogeneity (I^2^=65.24%, *p* = 0.009). In the sensitivity analysis, the heterogeneity was reduced to an acceptable level (I^2^=20.75%, *p* = 0.448) with the pooled result altered (pooled OR = 1.450, 95% CI: 1.009–2.083) after the study of Han and colleagues was removed, suggesting the pooled conclusion was not robust.

#### Use of vasopressor or inotrope at the cessation of CRRT

**Figure 9.**
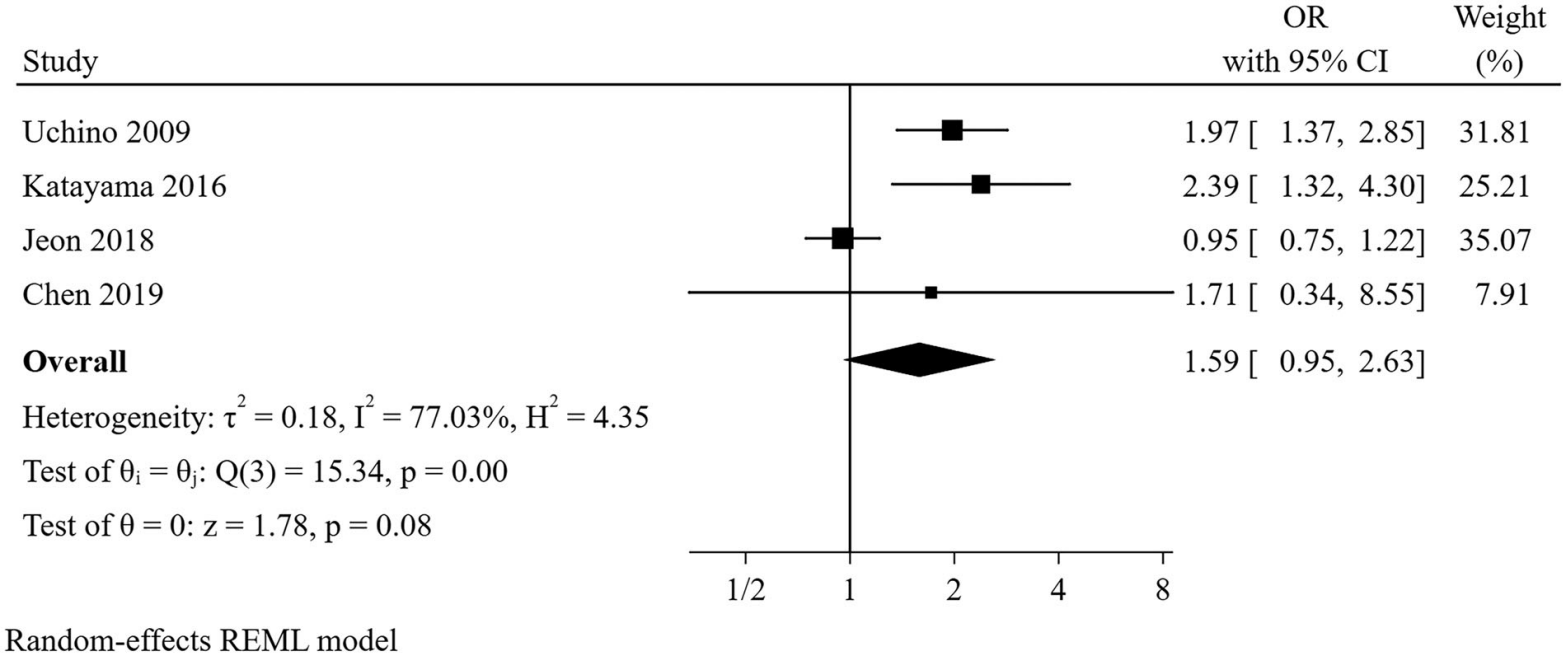
The forest plot showed the relationship between use of vasopressor or inotropes at the cessation of CRRT and successful weaning from CRRT.

Four studies [21,25,26,29] were included in the meta-analysis for use of vasopressor or inotrope at the cessation of CRRT (Figure 9). The result indicated that the use of vasopressor or inotrope at the cessation of CRRT was not a predictor for short-term successful weaning from CRRT (pooled OR = 1.586, 95% CI: 0.955–2.635). However, there was significant heterogeneity (I^2^=77.03%, *p* = 0.002). In the sensitivity analysis, the study of Jeon and colleagues was removed because the different definition of successful weaning from CRRT differs from other studies (3 days vs. 7 days), and the pooled result altered (pooled OR = 2.066, 95% CI: 1.521–2.805) without heterogeneity (I^2^=0%, *p* = 0.842), suggesting the pooled conclusion was not robust.

#### Use of diuretics at the cessation of CRRT

**Figure 10.**
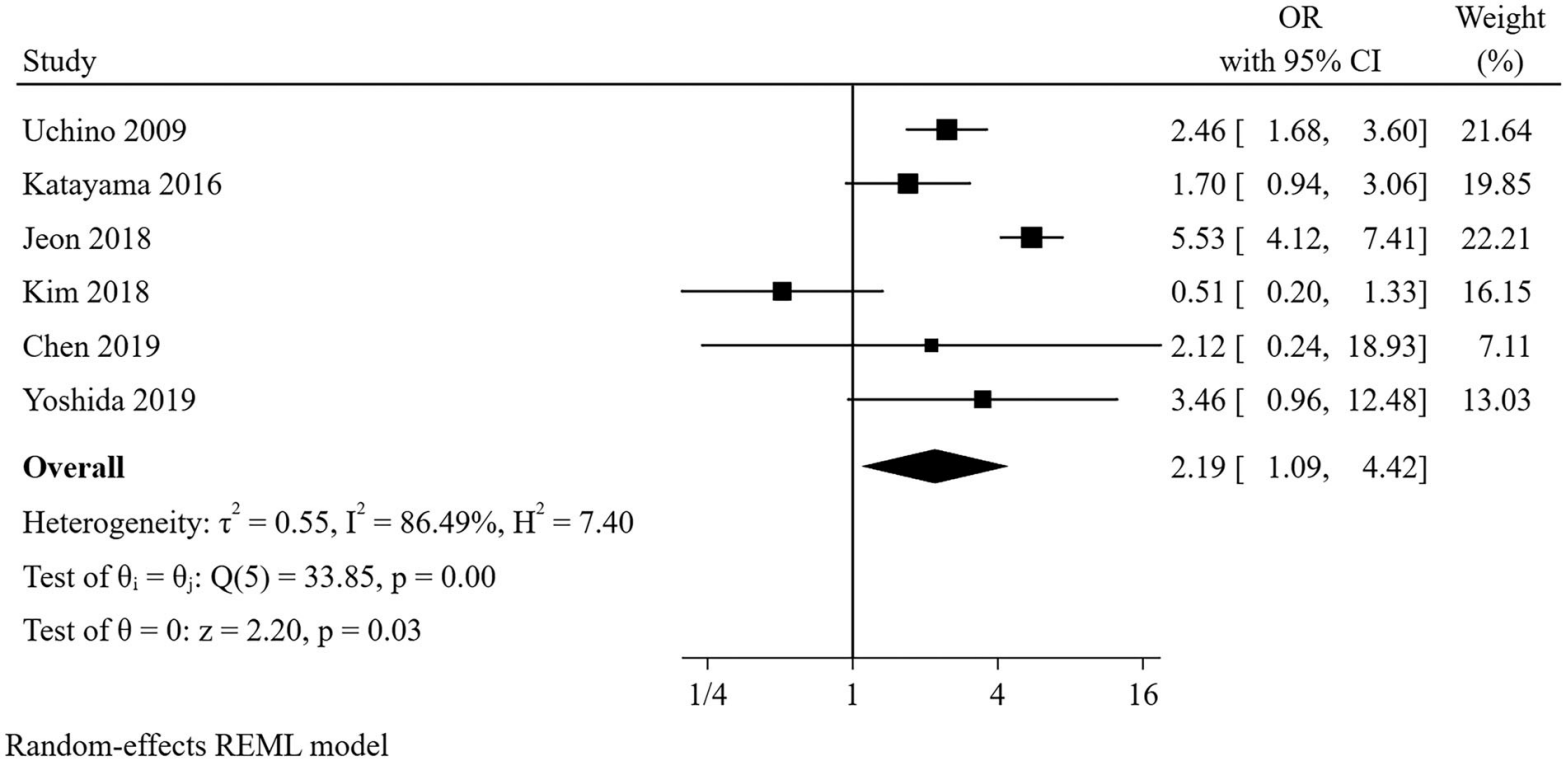
The forest plot showed the relationship between use of diuretics at the cessation of CRRT and successful weaning from CRRT.

Six studies [21,25–27,29,31] were included in the meta-analysis for use of diuretics at the cessation of CRRT (Figure 10). The result indicated that the use of diuretics at the cessation of CRRT was a predictor for short-term successful weaning from CRRT with significant heterogeneity (pooled OR = 2.195, 95% CI: 1.089–4.424, I^2^=86.49%, *p* < 0.001). Sensitivity analysis indicated the pooled conclusion was not robust. We didn’t find the source of heterogeneity through the subgroup analysis.

#### Urine output at the cessation of CRRT

**Figure 11.**
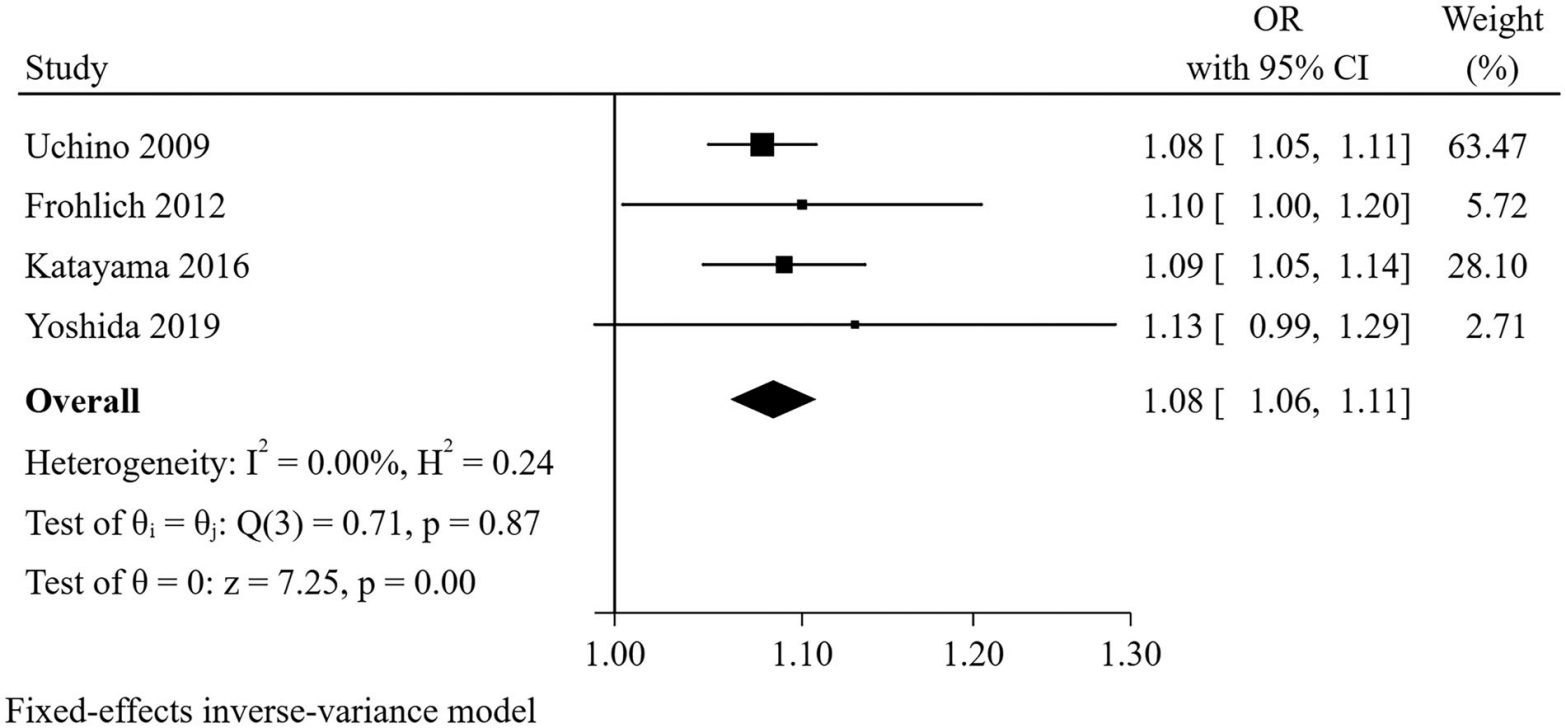
The forest plot showed the relationship between urine output at the cessation of CRRT and successful weaning from CRRT.

Four studies [21,22,25,31] were included in the meta-analysis for urine output at the cessation of CRRT (Figure 11). The result suggested that urine output at the cessation of CRRT (per 100 mL/day increase) was a predictor for short-term successful weaning from CRRT (pooled OR = 1.084, 95% CI: 1.061–1.108, I^2^=0%, *p* = 0.872). Sensitivity analysis indicated the pooled conclusion was robust.

#### Serum creatinine at the cessation of CRRT

**Figure 12.**
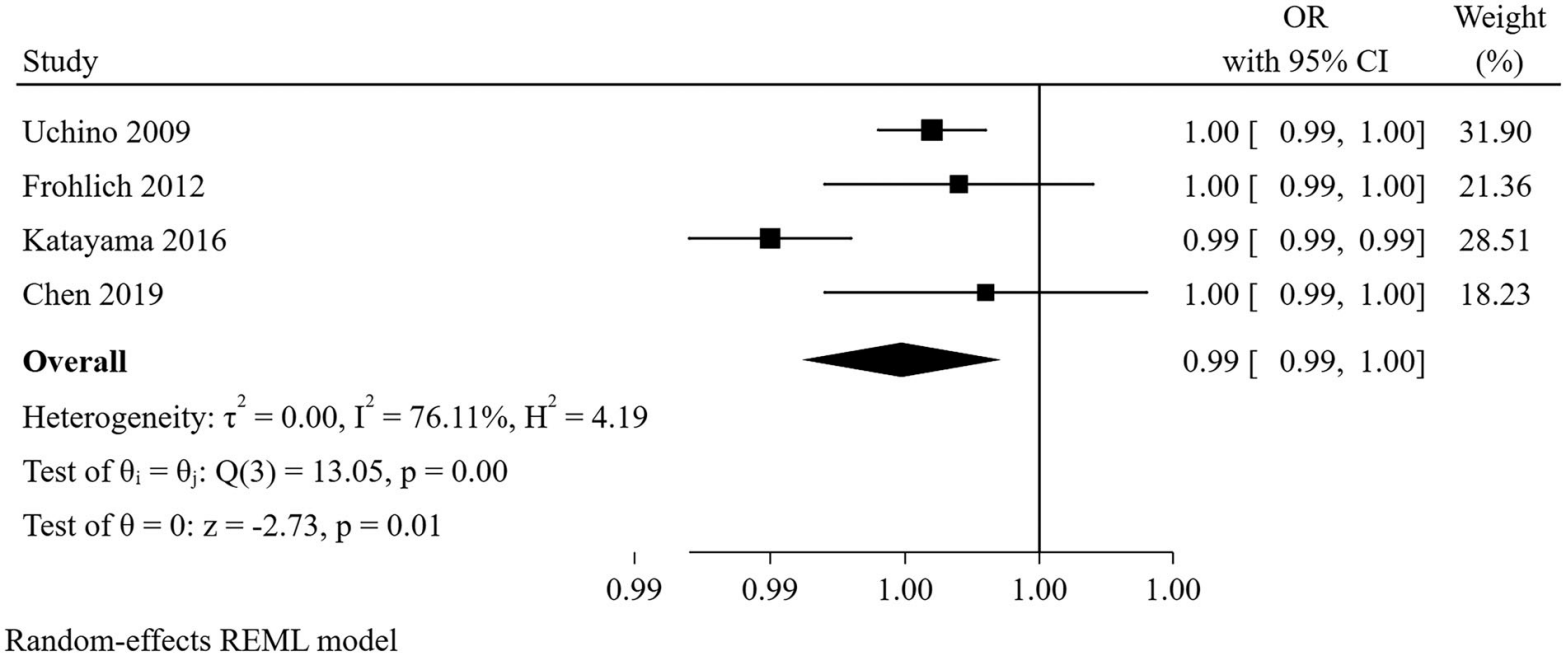
The forest plot showed the relationship between serum creatinine at the cessation of CRRT and successful weaning from CRRT.

Four studies [21,22,25,29] were included in the meta-analysis for serum creatinine at the cessation of CRRT (Figure 12). The result suggested that serum creatinine at the cessation of CRRT (per 1 μmol/L increase) was a predictor for short-term successful weaning from CRRT (pooled OR = 0.995, 95% CI: 0.991–0.999). However, there was a high degree of heterogeneity among the studies (I^2^=76.11%, *p* = 0.005). The heterogeneity disappeared after excluding the study of Katayama and colleagues in the sensitivity analysis, but the pooled result was not changed (pooled OR = 0.996, 95% CI: 0.995–0.998). Nevertheless, this result needs to be interpreted with caution due to the upper limit of the confidence intervals that did approach non-significance.

#### Others

Some variables could not be included in the meta-analysis because of missing data, consisting of fluid balance [30], serum cystatin C (CysC) [27,32], N-terminal prohormone of brain natriuretic peptide (NT-proBNP) [24], neutrophil gelatinase-associated lipocalin (NGAL) [27,29], kinetic estimated glomerular filtration rate (eGFR) [31], 2-h creatinine clearance [22], the Sequential Organ Failure Assessment (SOFA) score [23], and the Acute Physiology and Chronic Health Evaluation (APACHE) ІІ score [24].

## Discussion

The systematic review and meta-analysis evaluated 11 variables for short-term weaning from CRRT in severe AKI patients, and the results suggested that CKD, CRRT duration, and urine output (per 100 mL/day increase) at the cessation of CRRT were predictive factors. In a recent systematical review and meta-analysis, Katulka et al. [33] concluded that urine output prior to discontinuation of KRT was the most commonly described and robust predictor. However, the previous meta-analysis was performed with a search strategy combining the themes of kidney replacement therapy (IHD, CRRT, SLED) and there was considerable heterogeneity among studies (e.g., the definition of weaning success, variable heterogeneity, study design). Our study differs from the previous one in several respects. First, only patients with AKI undergoing CRRT were included. Second, the definition of short-term successful weaning from CRRT is free from KRT for 7 or 14 days after the cessation of CRRT for most of the included studies. Thirdly, in addition to urine output, clinical and biochemical parameters were also evaluated to enhance predictive accuracy in our study.

Regarding comorbidities, there is ample biological plausibility to support the role of CKD as a risk factor for AKI and the relationship has been demonstrated in different patient populations [34–36]. Patients with CKD are more likely to present with fluid overload because of decreased kidney function. This is well supported by our pooled results indicating that CKD was a strong negative predictor for successful weaning from CRRT (OR = 0.638), however, for the specific impact of CKD GFR and albuminuria categories, no further assessment was possible given the available data. In the intensive care unit setting, sepsis remains the primary cause of AKI and accounts for approximately half of all cases [2,37]. In septic AKI, 20–30% of patients progress to kidney failure and is associated with high mortality [38,39]. Our findings suggest that sepsis was not related to short-term successful weaning from CRRT. Whereas in the German study, the SOFA score, as an assessment method for evaluating the severity of organ dysfunction, has been shown to be associated with the discontinuation of CRRT in a multivariate model [23]. Because of the limited number of studies involved in SOFA score, we cannot perform a meta-analysis. As for diabetes mellitus, our results tend to toward not a predictor. Furthermore, for patients with hypertension, due to the heterogeneity, more studies would be necessary to verify the obtained result.

Among the variables studied, urine output was the most frequently investigated variable. But substantial heterogeneity was observed among studies in the threshold value for urine output during weaning from CRRT, ranging from 191 mL/day [26] to 1700 mL/day [31]. In addition, urine output weaning criteria are affected by diuretics, which are commonly used in intensive care unit patients. Some studies described the reduced predictive ability of urine output after the use of diuretics [28,31] and others showed a better discriminative power after the use of diuretics [21,26]. Recently, a meta-analysis pooled urine output to confirm the predictive ability for liberation from renal replacement therapy, with a pooled sensitivity of 66.2% and a pooled specificity of 73.6% [33]. However, the opposite conclusion for urine output at the cessation of CRRT (mL/h/kg) has also been reported by Kim and colleagues in a multivariable model (OR = 1.476, 95% CI: 0.942–2.314) [27]. At present, insufficient data are available to derive a threshold for urine output to accurately predict successful weaning from CRRT, and our results showed for the first time that urine output at the cessation of CRRT (per 100 mL/day increase) was an important predictor for successful weaning from CRRT. We expect that we will further improve the predictive ability of urine output by the combination of patient-specific variables (e.g., CRRT duration, CKD status), biomarkers (e.g., CysC, NGAL, kinetic eGFR), and clinical severity scores (e.g., SOFA score, APACHE II score).

The predictive accuracy of serum creatinine at the cessation of CRRT has been previously assessed [21,22,25,29]. According to our result, we are inclined to believe serum creatinine at the cessation of CRRT is a predictor for successful weaning from CRRT. However, our pooled analysis result was not robust with high heterogeneity. This could be due to the limited number of included studies, or the effect size of the change (per 1 μmol/L increase) is too small to explain the observed changes. In addition, serum creatinine levels are affected by many factors (e.g., muscle mass, gender, race, the timing of cessation of CRRT). As for the use of diuretics, we are inclined to believe that patients reduced demand on re-initiation of RRT with enhanced diuresis therapeutic strategy, due to less fluid accumulation after the termination of CRRT. However, the result should be carefully interpreted because of the significant heterogeneity among studies, and some of the heterogeneity may be due to the different types of diuretics and management strategies. It is commonly accepted that maintenance of appropriate blood pressure with vasopressor or inotrope brings clinical benefits, but more studies are needed to verify the relationship between drug use and short-term successful weaning from CRRT due to the heterogeneity.

The strengths of this study lie in the strict adherence to The Cochrane Library and PRISMA guidelines. Our study identified and synthesized a wide array of predictive variables on physiologic and biochemical parameters for short-term successful weaning for CRRT.

Despite these strengths, our study presents several limitations. First, due to the enrollment of cross-sectional studies in our meta-analysis, causal relationships could not be verified. Second, the definition of short-term successful weaning from CRRT varied among included studies, which may be a potential cause for the heterogeneity. In the present study, the successful discontinuation was mostly defined as free from CRRT for 7–14 days, while the successful discontinuation of KRT may be more important than that of CRRT in clinical practice. However, we could not obtain sufficient data to carry out a meta-analysis. Third, the decision for CRRT discontinuation depended on physicians’ judgment but not standardized criteria in the majority of included studies.

## Conclusions

The presented systematic review and meta-analysis indicated that CKD, CRRT duration, and urine output (per 100 mL/day increase) at the cessation of CRRT were predictors for short-term weaning from CRRT. As for the variables of hypertension, use of vasopressor or inotropes at the start or at the cessation of CRRT, use of diuretics at the cessation of CRRT, and serum creatinine at the cessation of CRRT, further large prospective studies are required to clarify this association.

## Ethical approval and consent to participate

Not applicable. No patients are discussed or were involved in this publication. All data sources are from previously published literature.

## Geolocation information

No. 201-209 Hubin South Road, Siming District, Xiamen, Fujian, China.

## Supplementary Material

Supplemental Material

## Data Availability

The authors confirm that the data supporting the findings of this study are available within the article.
