# Supplementary material for: Predictors for short-term successful weaning from continuous renal replacement therapy: a systematic review and meta-analysis
Source: Ren Fail. 2023 Feb 10;45(1):2176170. doi: 10.1080/0886022X.2023.2176170 (PMC9930790; doi:10.1080/0886022X.2023.2176170)
Supplement: Supplemental Material [file IRNF_A_2176170_SM4876.pdf]

**Additional file 1.** The complete search strategies used for PubMed, Embase, and the Cochrane Library databases

**1. Search strategy in PubMed and the Cochrane library:**

#1. (Continuous Renal Replacement Therapy [Mesh]) OR (Continuous RRT) OR (RRT, Continuous)

#2. (weaning) OR (discontinuation) OR (cessation)

#3. (risk) OR (risk*) OR (predict*) OR (associat*) OR (correlat*) OR (odds ratio*) OR (rate ratio*) OR (hazard ratio*)

#4. #1 AND #2 AND #3

**2. EMBASE:**

#1. 'continuous renal replacement therapy'/exp OR 'continuous rrt' OR 'RRT, Continuous'

#2. 'risk'/exp OR ' risk*' OR 'predict*' OR 'associat*' OR 'correlat*' OR 'odds ratio*' OR 'rate ratio*' OR 'hazard ratio*'

#3. 'weaning'/exp OR 'discontinuation' OR 'cessation'

#4. #1 AND #2 AND #3

Information Classification: General

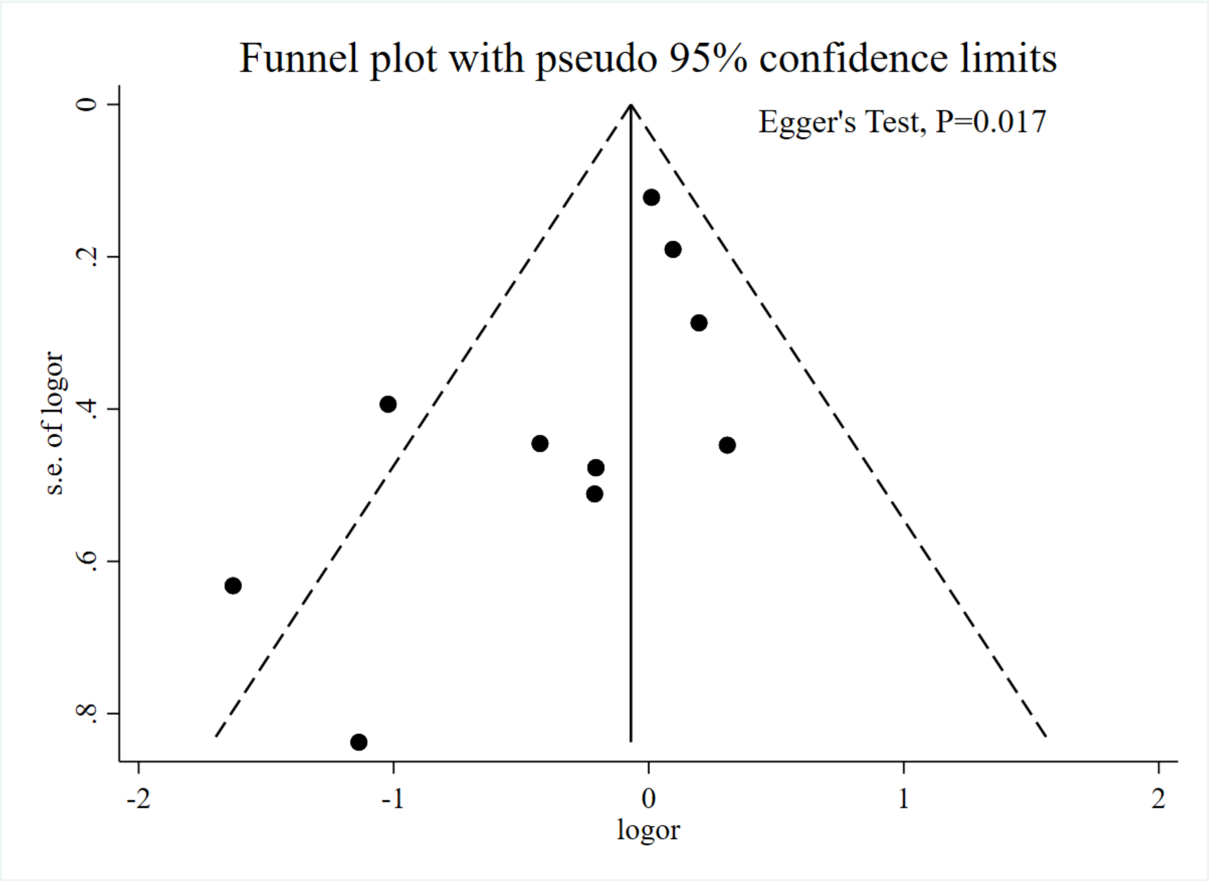


**Additional file 2.** The funnel plot and Egger's test for gender

Information Classification: General

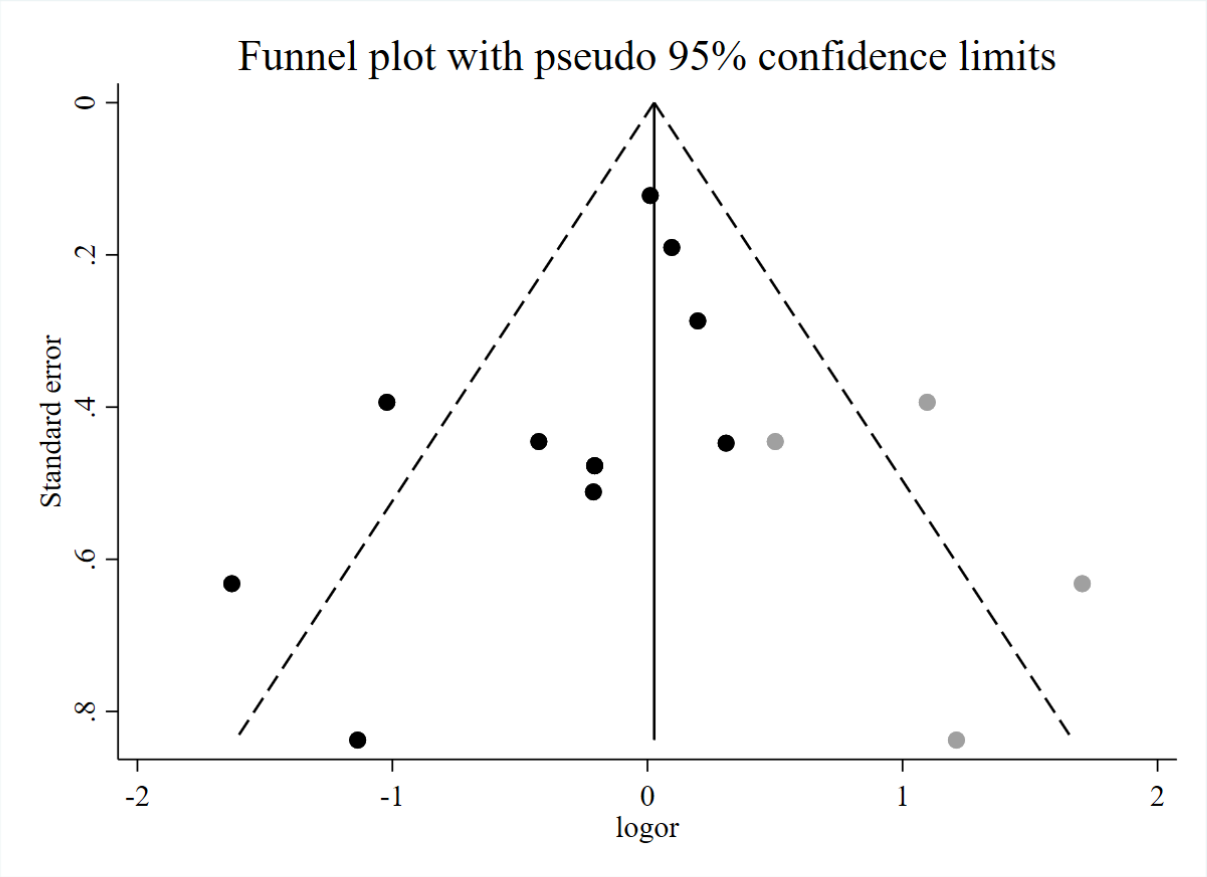


**Additional file 3.** The funnel plot using the trim and fill method for gender

Information Classification: General

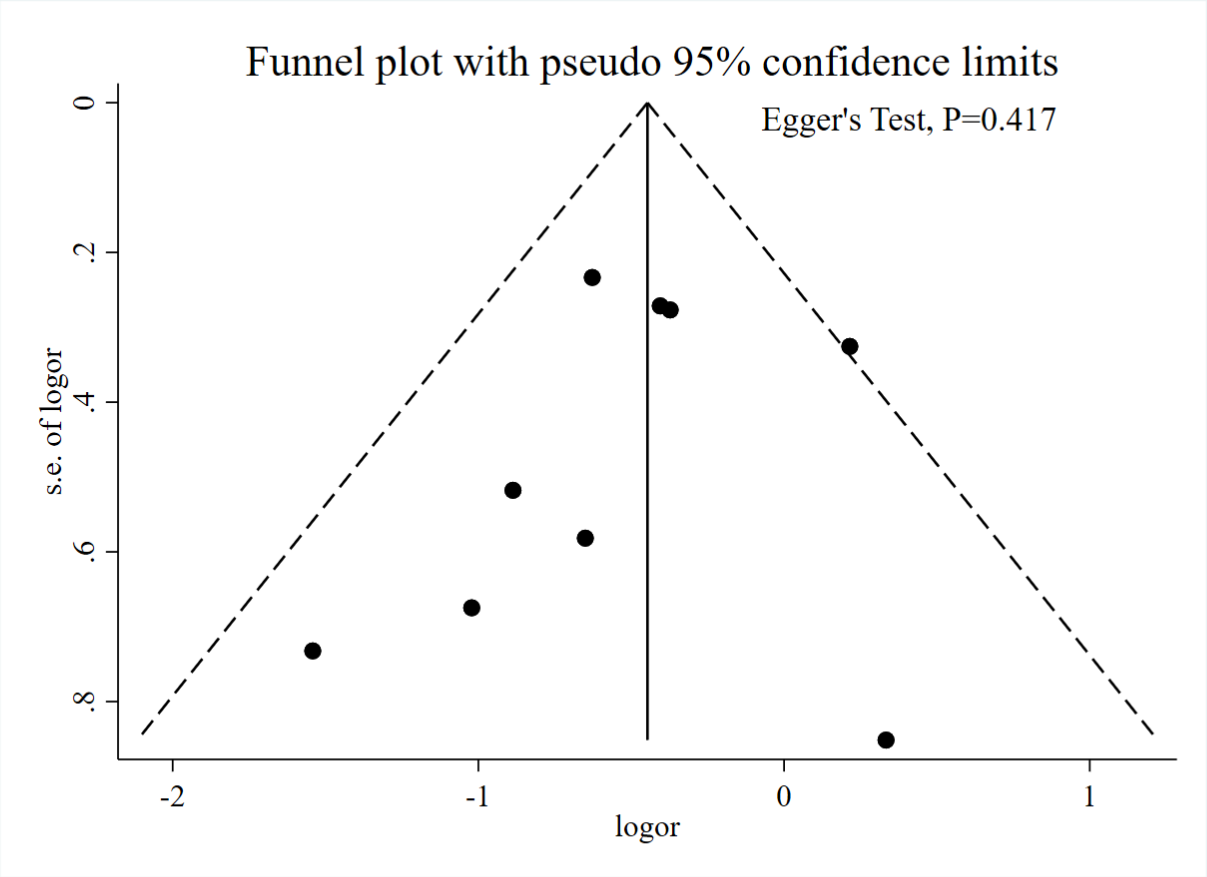


**Additional file 4.** The funnel plot and Egger's test for chronic kidney disease

Information Classification: General
